# Novel Therapies for Parkinsonian Syndromes–Recent Progress and Future Perspectives

**DOI:** 10.3389/fnmol.2021.720220

**Published:** 2021-08-26

**Authors:** Dominika Przewodowska, Weronika Marzec, Natalia Madetko

**Affiliations:** ^1^Students' Scientific Association of the Department of Neurology, Medical University of Warsaw, Warsaw, Poland; ^2^Department of Neurology, Medical University of Warsaw, Warsaw, Poland

**Keywords:** atypical parkinsonism, novel therapies, α-synuclein, tau-protein, disease-modifying, multiple system atrophy, progressive supranuclear palsy, corticobasal degeneration

## Abstract

**Background:** Atypical parkinsonian syndromes are rare, fatal neurodegenerative diseases associated with abnormal protein accumulation in the brain. Examples of these syndromes include progressive supranuclear palsy, multiple system atrophy, and corticobasal degeneration. A common clinical feature in parkinsonism is a limited improvement with levodopa. So far, there are no disease-modifying treatments to address these conditions, and therapy is only limited to the alleviation of symptoms. Diagnosis is devastating for patients, as prognosis is extremely poor, and the disease tends to progress rapidly. Currently, potential causes and neuropathological mechanisms involved in these diseases are being widely investigated.

**Objectives:** The goal of this review is to summarize recent advances and gather emerging disease-modifying therapies that could slow the progression of atypical parkinsonian syndromes.

**Methods:** PubMed and Google Scholar databases were searched regarding novel perspectives for atypical parkinsonism treatment. The following medical subject headings were used: “atypical parkinsonian syndromes—therapy,” “treatment of atypical parkinsonian syndromes,” “atypical parkinsonian syndromes—clinical trial,” “therapy of tauopathy,” “alpha-synucleinopathy treatment,” “PSP therapy/treatment,” “CBD therapy/treatment,” “MSA therapy/treatment,” and “atypical parkinsonian syndromes—disease modifying.” All search results were manually reviewed prior to inclusion in this review.

**Results:** Neuroinflammation, mitochondrial dysfunction, microglia activation, proteasomal impairment, and oxidative stress play a role in the neurodegenerative process. Ongoing studies and clinical trials target these components in order to suppress toxic protein accumulation. Various approaches such as stem cell therapy, anti-aggregation/anti-phosphorylation agent administration, or usage of active and passive immunization appear to have promising results.

**Conclusion:** Presently, disease-modifying strategies for atypical parkinsonian syndromes are being actively explored, with encouraging preliminary results. This leads to an assumption that developing accurate, safe, and progression-halting treatment is not far off. Nevertheless, the further investigation remains necessary.

## Introduction

The term “atypical Parkinsonian syndromes” (APS) refers to chronic progressive neurodegenerative diseases with a common primary feature, parkinsonism with a poor or waning levodopa response and coexistence of additional “plus” features, hence, also often referred to as Parkinson-plus disorders. Progressive supranuclear palsy (PSP), multiple system atrophy (MSA), and corticobasal degeneration (CBD) can be distinguished among them. A shared pathogenetic feature of these disorders is abnormal protein accumulation in different brain regions. PSP and CBD are neuropathologically described as four-repeat tauopathies, conditions characterized by deposition of phosphorylated tau protein in neurons and glia, leading to toxicity and cell loss. Alpha-synuclein protein, the primary component of Lewy bodies, is also found in glial cytoplasmic inclusions characteristic of MSA (Dickson, 2012; Levin et al., 2016). APS are severe and tend to progress rapidly. Diagnosis of APS may be difficult, especially at the early stages of the disease, because of overlapping clinical manifestation with PD. This can have detrimental consequences considering that prognosis and life expectancy are substantially worse among atypical parkinsonian disorders (McFarland, 2016). Despite increasing knowledge of the neuropathological mechanisms of these disorders, there are no available disease-modifying treatments for APS, and current therapy is limited to alleviating clinical symptoms. Each example of APS is an orphan disease, which, according to its definition, affects no more than 1 in 2000 people in the European population or is neglected. Because of limited financial incentives, APS-targeted treatments have long been ignored in the pharmaceutical market. With recent advances in research, changing demographics, and the increasing prevalence of APS in the aging population, APS-targeted interventions are gaining the attention of pharmaceutical companies, which brings hope for novel therapeutic approaches.

Studies assessing the use of dopaminergic drugs in the pharmacotherapy of atypical parkinsonism have shown ambiguous results. While some describe modest clinical improvement, the general therapeutic effect is rather insignificant (Kuran, 2007; Greene, 2019). For example, some studies suggest that levodopa responsiveness is a sign of a more beneficial course of MSA (Ishida et al., 2021). The use of dopamine agonists, such as rotigotine, in PSP was shown to possibly preserve some cognitive abilities (Schirinzi et al., 2019). However, only 6 out of 7 participants completed this study, and its protocol did not include placebo-controlled group. Another clinical trial showed improvement after pramipexol administration among MSA patients with no response to levodopa; however, this is only a single report (Ueda et al., 2013). In a study evaluating the effectiveness of amantadine (100 mg twice daily) in PSP and MSA, 42.9% of the PSP patients and 61.5% of the MSA patients showed partial improvement (Rajrut et al., 1997). A more recent *post-hoc* analysis of the role of amantadine (227.9 mg daily) in PSP did not show any positive correlation between the use of amantadine and cognition or gait efficiency (Dale et al., 2020). Atypical parkinsonism symptoms were also evaluated following treatment with the monoaminoxidase inhibitor rasagiline, showing neuroprotection in transgenic MSA models (Stefanova et al., 2008). However, in a 48-week study examining 174 participants, no therapeutic efficacy was shown in humans with daily administration of 1 mg rasagiline as measured by the Unified Multiple System Atrophy Rating Scale (Poewe et al., 2015).

**Figure 1:**
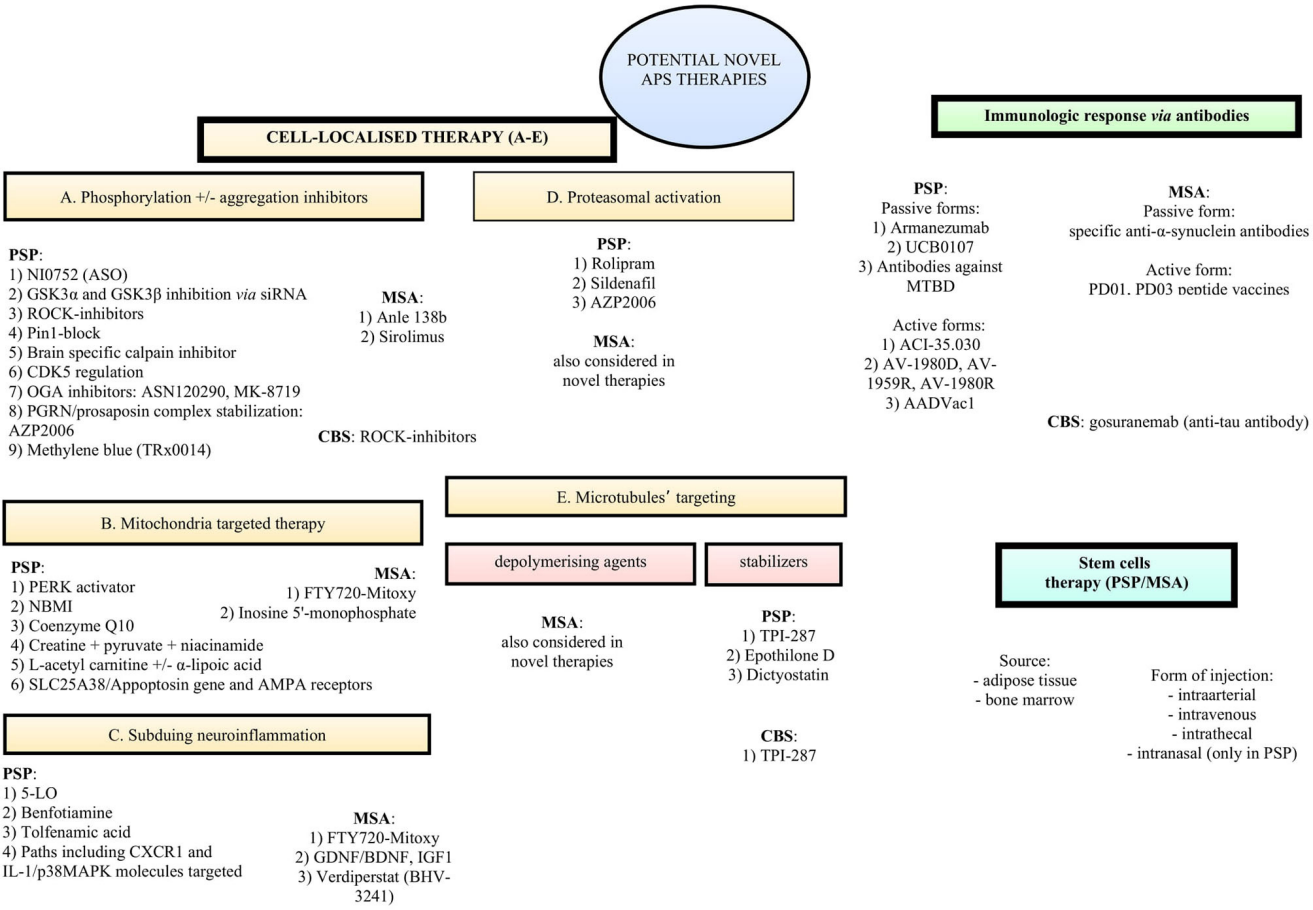
Potential novel therapies for atypical parkinsonian syndromes.

Since there is no disease-modifying treatment for progressive supranuclear palsy, therapy is currently focused on relieving clinical symptoms. Current approaches include botulinum toxin A (BtxA) injections used in focal dystonia (Müller et al., 2002) and a combination of levodopa and dopamine agonists, which may mildly improve motor symptoms such as bradykinesia and hypokinesia, tremor, and gait impairment, but usually only at the beginning of the disease (Birdi et al., 2002). Education about safe swallowing, balance-keeping, and strategies for proper falling are important protective approaches for patients to minimize the risk of further complications and accidents (Agarwal and Gilbert, 2020). Nevertheless, searching for disease-modifying treatments is of great scientific interest for many groups. Thus, this review summarizes recent progress and perspectives regarding novel therapies for APS (Figure 1).

## Progressive Supranuclear Palsy

Progressive supranuclear palsy is an akinetic-rigid form of parkinsonism caused by intracerebral accumulation of the hyperphosphorylated microtubule-associated protein tau (MAPT). Abnormal aggregation of tau, a microtubule-binding protein, results in defective microtubule activity, a significant feature of this disease (Liu and Gong, 2008). The 4R-form of tau is most prevalent pathologically and is morphologically defined as neurofibrillary tangles and tufted astrocytes (Borroni et al., 2011; Höglinger et al., 2017; Agarwal and Gilbert, 2020). Due to this pathology, modification of tau protein is a potential therapeutic in PSP treatment (Schneider and Mandelkow, 2008). The clinical phenotype of PSP varies. Moreover, there is no histological basis providing the correct and accurate distinction between different PSP phenotypes, excluding assessment of the distribution of tau aggregates (Dickson et al., 2010; Agarwal and Gilbert, 2020). Diagnosing PSP as early as possible seems to play a significant role not only in estimating the prognosis of the patient but also in conducting innovative therapeutic trials (Borroni et al., 2011). Ongoing research is focused on potential disease triggers, such as oxidative stress and genetic mutations, since the primary cause of the disease remains unknown (Rampello et al., 2005; Borroni et al., 2011).

### Epidemiology

The prevalence of progressive supranuclear palsy is estimated by different studies to be approximately 6 per 100,000 (Schrag et al., 1999; Kawashima et al., 2004), with a general trend to increase with age from 1.7 cases per 100,000 people aged 50–59 to 14.7 per 100,000 in people aged 80–89 (Agarwal and Gilbert, 2020). The average time of disease-onset is 65-69 years (Coyle-Gilchrist et al., 2016) with male predominance globally (Bower et al., 1997).

### Treatment

Novel PSP therapeutic approaches are focused on slowing or halting disease progression, beyond only managing its physical, behavioral, and emotional symptoms. Recent studies have focused on treatment at the molecular level, not only examining genes responsible for tau protein synthesis and aggregation but also controlling molecules that participate in the degradation of misfolded-tau and prevention of oxidative stress (Boxer et al., 2017). Another approach that has the potential to be effective in PSP is the transfer of autologous mesenchymal cells derived from bone marrow (US National Library of Medicine, 2016) or adipose tissue (Choi et al., 2014).

### Targeting Inflammation

Microglia are the most important immune modulator in the human nervous system (Maphis et al., 2015); these cells release cytokines crucial for processes related to nervous system activity and immune responses (Hanisch, 2002). They also have a capacity to respond to neuronal stimulation, leading to increased expression of receptors for glutamate and γ-aminobutyric acid (GABA) (Färber and Kettenmann, 2005).

*In vivo* studies suggest that microglial activity leads to the activation of p38-mitogen activated protein kinase (p38-MAPK), which triggers phosphorylation and aggregation of tau protein (Maphis et al., 2015). In addition, tau accumulation appears to be connected with the expression of microglial-specific fractalkine receptor (CX3CR1), toll-like receptor 4 (TLR4), and receptors activated by interleukin-1 (IL-1R). The authors suggest that these proteins, such as CX3CR1 and IL-1/p38MAPK, may be novel targets for human tauopathy treatment (Bhaskar et al., 2010).

Inflammation may also play a role in PSP, as it is commonly associated with tauopathies, despite limited evidence assessing the pathologic connection (Vagnozzi et al., 2017). 5-Lipooxygenase (5-LO) is a critical enzyme in the onset of inflammation that acts by inducing leukotrienes (LTs) activation (Kim et al., 2008) and is broadly expressed in the central nervous system (Vagnozzi et al., 2017). Trials conducted on an Alzheimer's disease (AD) Tg2576 mouse model with overexpression of the 5-LO gene showed not only increased level of biomarkers, indicating astrocytes and microglia activation [by elevation of glial fibrillary acidic protein (GFAP) and CD45 concentration], but also enhanced amyloid-β (Aβ) aggregation (Chu et al., 2012). Further investigations performed on tau transgenic mice demonstrated a significant effect of 5-LO inhibition, resulting in a reduced number of tau aggregates and cognitive improvement. Mechanisms of tau elimination were correlated with cyclin-dependent kinase 5 (CDK5) activation (Giannopoulos et al., 2015).

A case-control study verifying the relationship between nonsteroidal anti-inflammatory drug (NSAID) intake and progressive supranuclear palsy symptoms showed no meaningful results. The authors emphasized that in order to obtain information on the correlation between NSAID use and change in the severity of PSP symptoms, a larger study should be conducted (Marras et al., 2018).

Reduction of thiamine and thiamine-dependent enzyme activity resulted in tau phosphorylation, Aβ accumulation, and oxidative stress exacerbation, leading to increased interest in benfotiamine (BFT) as a potential PSP therapy (Tapias et al., 2018). BFT is a synthetic lipophilic derivative of thiamine (Raj et al., 2018). BFT reduces amyloid aggregates, decreases tau levels, and activates the phosphorylation of glycogen synthase kinase-3α and 3β (GSK-3α/β), leading to attenuation of its activity in AD transgenic mice (Pan et al., 2010). Thiamine deficiency is an important factor in oxidative stress regulation, since it has been shown to cause progression of neurodegeneration and, subsequently, has become a target for future therapies (Lin and Beal, 2006; Tapias et al., 2018). Thiamine deficiency activates microglia, boosts reactive oxygen species (ROS) production, and facilitates blood brain barrier (BBB) damage (Calingasan et al., 1998). Although microglial cells have a protective neuronal effect, they may also produce an enormous amount of chemokines involved in neuronal injury response (Gyoneva and Ransohoff, 2015). Dietary BFT in P301S tau transgenic mice showed positive effects on physical outcomes and decreased levels of glycated tau. BFT treatment triggers NF-E2-related factor 2/antioxidant responsive element (Nrf2/ARE) activation, resulting in cellular metabolic rearrangement. This includes decreases in inflammation markers cyclooxygenase-2 (COX-2), tumor necrosis factor α (TNFα), and proteins associated with nitration and peroxidation processes (Tapias et al., 2018). More recently, BFT has been identified as a modulator of microglia-inhibition with anti-inflammatory facilities, such as the alleviation of heat-shock protein 70 (Hsp70) and COX-2 release. Because of its ability to block kinases activity and manage NF-κB transport, BFTmay have neuroprotective properties (Bozic et al., 2015).

Another drug believed to be important for future PSP treatment is tolfenamic acid (TA). Classified as an NSAID, TA is effective in pain reduction and lowering body temperature, and it exhibits characteristics of a possible anti-neoplastic molecule (PubChem, 2021). Recent studies demonstrated that TA alters tau phosphorylation and reduces total tau distribution in the mouse central nervous system, causing memory improvement (Chang et al., 2018). Future results of a phase 2a trial evaluating the safety and efficacy of TA oral intake (50, 300, or 600 mg daily, compared with placebo) in patients with PSP could provide evidence for the successful application of TA in PSP therapy. The safety of TA treatment will be measured by the number of adverse events, changes in ECG, nasal examination, and clinical laboratory tests over 12 weeks. The trial is planned to include 24 participants and to be completed by December 31, 2022 (US National Library of Medicine, 2020a).

### Modulation of Oxidative Stress

Recently published data have highlighted the significance of mitochondrial dysfunction and oxidative stress in PSP. Studies on cells expressing mitochondrial genes found in patients with PSP revealed decreased activity of mitochondrial complexes I and III involved in ATP production and significant increases in antioxidant enzyme activity and markers of lipid oxidative damage, suggesting oxidative injury (Albers et al., 2001; Chirichigno et al., 2002; Stamelou et al., 2010). Single nucleotide polymorphism (rs1768208 C/T) located near the myelin-associated oligodendrocyte basic protein (MOBP) gene and related to the SLC25A38/appoptosin gene is also a genetic variant connected with PSP outcome (Zhao et al., 2015). Appoptosin is a member of a family of mitochondrial proteins located in the inner mitochondrial membrane involved in molecular transport (Haitina et al., 2006; Ogunbona and Claypool, 2019). Appoptosin is also critical for heme synthesis (Guernsey et al., 2009), and its overexpression leads to exorbitant heme production, which results in disruption of homeostasis, increased level of ROS, and damage to endothelial cell structure. This disrupts the mitochondrial membrane potential and causes oxidative stress. Even minimal fluctuation in appoptosin can lead to cytoskeletal lability and inflammation (Kumar and Bandyopadhyay, 2005; Zhang et al., 2012b). Appoptosin expression in transgenic mice leads to tau accumulation, resulting in motor impairment and altered synaptic structures (Zhao et al., 2015). Another consequence of SLC25A38/appoptosin upregulation is the transfer of cytochrome C to the cytoplasm and both caspase-3 and caspase-9 activation, which are responsible for cell death (Zhang et al., 2012b; Brentnall et al., 2013). Caspase-3 activation also results in decreased α-amino-3-hydroxy-5-methyl-4-isoxazolepropionic acid (AMPA) and N-methyl-D-aspartic acid (NMDA) receptors in postsynaptic membranes (Zhao et al., 2015). AMPA receptors are, thus, a potential target for the treatment of neurodegenerative diseases, autism, and drug addiction (Lee et al., 2016).

Co-enzyme Q10 is a cofactor that stabilizes the mitochondrial respiratory chain and provides antioxidant properties (Saini, 2011). The ratio between Q10 in its reduced and oxidized form is a measure of oxidative stress level (Lagendijk et al., 1996). Two trials investigating the impact of Q10 in patients with PSP led to ambiguous conclusions. During the shorter trial (6 weeks), 5 mg/kg of daily Q10 was safe and well-tolerated, and increased metabolic activity and significantly improved clinical scores as measured by the Progressive Supranuclear Palsy Rating Scale (PSPRS) and Frontal Assessment Battery (FAB) (Stamelou et al., 2008). Another Q10 trial over a period of 12 months showed no statistically significant differences between 2,400 mg/d Q10 dose and placebo intake; however, there was a trend toward modest improvement (Apetauerova et al., 2016).

Niacinamide is a form of vitamin B3, which, along with tryptophan, serves as a source of nicotinamide adenine dinucleotide (NAD+) and nicotinamide adenine dinucleotide phosphate (NADP+), and their reduced forms (Makarov et al., 2019). Studies show that NAD+ is an important cofactor in metabolic redox reactions, and that it is involved in the regulation of cell death processes (Dölle et al., 2013). A clinical trial (registered as NCT00605930) on the safety and tolerance of a 6-month treatment with creatine, pyruvate, and niacinamide assessed their ability to penetrate the BBB, as measured by their metabolite concentrations in the cerebrospinal fluid (CSF). Unfortunately, study results were not published (US National Library of Medicine, 2008–2017; Shoeibi et al., 2018).

α-Lipoic acid and L-acetyl carnitine, administered together, are thought to have a potential benefit for patients with PSP. This combination demonstrated neuroprotective properties in a rotenone-induced PD mouse model. Another study conducted with young and old rats supplemented with acetyl-L-carnitine (ALCAR) showed that ALCAR rescued age-related mitochondrial dysfunction, maintained inner mitochondrial membrane stability, and contributed to a decrease in antioxidant production (Hagen et al., 1998). However, a human trial limited to 11 subjects using 600 mg/1.5 g α-lipoic acid and L-acetyl carnitine intake over a 6-month period showed that the most frequent adverse effects were restlessness, seizures, insomnia, and dizziness (US National Library of Medicine, 2012–2017). The results considering changes in cerebral oxidative stress markers remain unpublished.

N,N'-bis (2-mercaptoethyl) isophthalamide acts as an antioxidant and heavy metal chelator (Secor et al., 2011), as it has high affinity for Hg^2+^, Pb^2+^, and Cd^2+^ (Clarke et al., 2012). An N,N'-bis (2-mercaptoethyl) isophthalamide (NBMI) trial, currently recruiting for its 2a phase, is planned to examine the effect of NBMI on motor and non-motor symptoms of PSP as measured by the PSPRS and Non-Motor Symptoms Scale [NMSS], atrophy (by MRI), and neurocognitive symptoms, such as depression and fatigue (US National Library of Medicine, 2019c).

### RNA Modulation

Antisplicing oligonucleotides are a method of ribonucleic acid modulation and are increasingly being used to regulate protein expression in a multitude of diseases (i.e., from Alzheimer's disease to progressive supranuclear palsy, targeting tau). Antisplicing oligonucleotides (ASOs) inhibit tau accumulation and stabilize the hairpin RNA structure (Boxer et al., 2017). Pre-mRNA, the product of tau gene transcription, is present in neuronal axons. Altered splicing of exon 10 of tau RNA leads to dysregulation and change in the ratio of 3 or 4 microtubule binding domains (Rajrut et al., 1997). Many MAPT point mutations lead to hairpin destabilization first, followed by the splicing of exon 10 (containing missense mutation in the mature protein), causing 3R-tau/4R-tau imbalance (Liu and Gong, 2008; Boxer et al., 2017). When tau is not connected to microtubules, it is prone to hyperphosphorylation and accumulation into neurofibrillary tangles (Liu and Gong, 2008; Höglinger et al., 2017). An increased level of 4R tau is linked to more severe seizures and other behavioral abnormalities in mice expressing human tau (Schoch et al., 2016). Both *in vitro* and *in vivo* studies show that MAPT ASOs significantly reduce human tau protein levels and neuronal loss (DeVos et al., 2017), acting as a protective factor against seizures in an adult mouse model (DeVos et al., 2013).

There is only one registered clinical trial investigating the safety, tolerability, and pharmacokinetic parameters of the intrathecally administered ASO called NIO752 in patients with progressive supranuclear palsy (NCT04539041). This trial will assess the adverse effects NIO752 and occurrence of suicidal behaviors by CSF sampling. The study has already started recruiting participants, but finalization of the trial is estimated to be October 17, 2023 (US National Library of Medicine, 2020g).

Hairpin short-interfering RNA (siRNA) inhibits both 3α- glycogen synthase kinase and 3β-glycogen synthase kinase (GSK-3α and GSK-3β), resulting in increased levels of β-catenin (Yu et al., 2003), a protein functioning as a homeostasis protector (MacDonald et al., 2009). Simultaneous implementation of two hairpin siRNA expression vectors, which is done in this study, will provide important information on the development of new treatment combinations for tauopathies (Yu et al., 2003; Shoeibi et al., 2018).

### Kinases and Enzymes Modulation

Defects in mitochondrial DNA can lead to disruption of the electron transport chain and exacerbation of oxidative stress, which contributes to increased activity of kinase pathways (Swerdlow et al., 2000; Ferrer et al., 2001). This phenomenon has been shown in PSP neurons and glial cells (Ferrer et al., 2001), and coincides with both detachment of tau from microtubules and accumulation of tau (Iqbal et al., 2008; Stamelou et al., 2010). As a result, kinase inhibition is considered as an important element in future PSP treatment.

Phosphorylated endoplasmic reticulum kinase (PERK) controls cellular response against unfolded or incorrectly folded proteins. Immunostaining studies have described a type of p62 and ubiquitin-positive aggregates, where p62 is a marker of autophagy dysfunction. Vesicles containing p62, ubiquitin, and microtubule-associated proteins 1A/1B light chain 3B (LC3) are evidence of lysosomal impairment (Bruch et al., 2015). PERK is the cellular protector against protein disruption associated with oxidative stress. There is evidence that there are different variants of PERK. For example, the allele connected with tauopathy is linked to decreased PERK activity, impairment of unfolded protein response (UPR), and accumulation of neurofibrillary tangles (Bruch et al., 2015; Yuan et al., 2018). By phosphorylating eukaryotic translation initiation factor 2A (EIF2A), PERK stimulates the activating transcription factor 4 (ATF4), a molecule involved in phagosome creation and minimizing mitochondrial destruction (Bouman et al., 2011; Yuan et al., 2018). Another gene activated by PERK is Nrf2, which is involved in oxidative stress regulation and is crucial for cell survival by mechanisms controlled by this kinase (Cullinan et al., 2003; Kansanen et al., 2013).

Both *in vitro* and *in vivo* studies conducted on male wild-type mice showed a significant effect of the pharmacological PERK activator, CCT020312 (selective eIF2a/PERK activator). This treatment provided neuroprotection, a decreased level of phosphorylated tau, and motor and cognitive improvements in a P301S tau mouse model (Bruch et al., 2017).

Rho-associated coiled-coil-containing protein kinases 1 and 2 (ROCK 1/2) are serine/threonine kinases involved in cellular motility (Riento and Ridley, 2003). Since it was demonstrated that the levels of ROCK1/2, p70 S6-kinase, and mTOR are increased in PSP and CBD brains, it led to the assumption that targeting of Rho kinases could be helpful in neurodegeneration treatment (Gentry et al., 2016). A clinical trial using Fasudil, an oral ROCK inhibitor, is already recruiting patients for a phase 2 investigation. The trial will include 15 participants, and will be conducted for 48 weeks with regular adverse effect controls, e.g., physical examinations, MRI, and laboratory tests measuring not only tau concentration but also neurodegeneration biomarkers, such as neurofilament light chain (NfL). Study completion date is estimated to be July 30, 2022 (US National Library of Medicine, 2021).

Another approach, based on genetic modifications, is altering the chemical structure of the tau protein. Studies focusing on traumatic brain injury (TBI), known as a risk factor for chronic traumatic encephalopathy (CTE) and AD, showed that neuronal damage results in an increased ratio of the cis-form of tau, which impairs axonal transport and leads to apoptosis. The whole phenomenon was called “cistauosis” (Kondo et al., 2015). Mouse studies showed that blocking the cis form of tau by specific antibodies decreases neurotoxicity and reduces behavioral defects. If tau acts as a protective factor against neuronal death, the greater understanding of tau could lead to changes in histological evaluation (Kondo et al., 2015).

As shown previously, the activity of peptidyl-prolyl cis-trans isomerase NIMA-interacting 1 (Pin1) protein is decreased in AD. Thus, the upregulation of this protein may be considered as a potential target for tauopathy treatment (Kondo et al., 2015). It has been shown that Pin1 preserves neurons and exerts a neuroprotective effect by modifying the chirality of tau and transforming it from its toxic cis- to trans–conformation (Ghosh et al., 2013). Increased amounts of cis-tau lead to microtubules aggregation, cis p-tau accumulation in other neurons, and improper axonal propagation (Ghosh et al., 2013). This suggests that monoclonal antibodies, which offset cis-tau toxicity, could be a promising therapy. This approach is already being used in trials focusing on Pin1 mechanisms in transgenic mouse models (Nakamura et al., 2012).

There are several candidate compounds analyzed in preclinical studies in order to effectively block tau phosphorylation, e.g., brain-specific calpain inhibitor and serine/threonine protein kinase 5 (CDK5) (Tyer and Hill, 2020). Calpain is a calcium-dependent protease taking part in cell death regulation (Cullinan et al., 2003). It participates in the degradation of cellular structures, such as membrane ion channels, adhesion molecules, receptors; and cytoskeletal proteins such as neurofilaments, α-fodrin, and lamins A and B located in the nucleus (Momeni, 2011; Silva and Haggarty, 2020).

CDK5 is involved in the regulation of the cell cycle and supports the creation of new synapses and vessels (Riento and Ridley, 2003). In studies with AD mouse models, it was shown that CDK5, by triggering destabilization of Bcl2-associated athanogene (BAG), leads to impairment of the Hsp70 system. This results in increased, unselective protein degradation and defects in glutamate-dependent paths. Improved cognitive effects from BAG3 expression are evidence for regulating CDK5 activity as a novel method of PSP therapy (Shupp et al., 2017; Zhou et al., 2020).

Blocking β-*N*-acetylglucosaminidase (O-GlcNAcase, OGA) is another potential method used to decrease the number of tau deposits. OGA is a glycoside hydrolase, providing breakdown of O-linked β-N-acetylglucosamine (O-GlcNAc) from proteins (Wells et al., 2002). O-GlcNAc participates in signaling pathways and regulation of protein activity by targeting their expression or degradation (Hart et al., 2007). O-GlcNAcylation of tau decreased the level of phosphorylated tau in both *in vitro* and *in vivo* studies (Liu et al., 2004). Mouse studies show that O-GlcNAc attachment to tau inhibits its aggregation and suppresses neuronal atrophy (Yuzwa et al., 2012). By targeting p53, NF-κB, and KEAP1/NRF2 pathways, O-GlcNAc also contributes to cellular oxidative stress level (Chen et al., 2018).

ASN120290, an inhibitor of O-GlcNAcase, was assessed in pre-clinical and clinical trials. In mice carrying P301L tau mutation, the inhibition of more than 80% of O-GlcNAcase activity led to a measurable increase in O-GlcNAcylated proteins in the brain (Hastings et al., 2017). The first phase of a human ASN120290 trial demonstrated that the drug was indeed safe, and that its pharmacokinetic parameters showed similar drug concentrations in CSF and plasma. There were no adverse effects reported, and no patients were withdrawn from the trial [ALZFORUM. (n.d.)]. In a recently announced trial conducted on healthy volunteers, neuronal ASN120290 distribution will be measured by positron emission tomography (PET). The goal of the study is to assess data on drug dosage for future PSP treatment (Asceneuron, 2018).

By magnetic resonance imaging, it was found that MK-8719, another O-GlcNAcase inhibitor, reduced tau aggregates and forebrain atrophy in transgenic tau rTg4510 mice (Wang et al., 2020). A phase 1 study has shown that the drug is well-tolerated, and that the plasma concentration of MK-8719 was proportional to the dose (only in oral intake between 5 and 600 mg) (Sandhu et al., 2016). In contrast, substances that showed great potential in treating PSP such as salsalate (an NSAID that targets acetyltransferase p300 and inhibits tau acetylation) (Min et al., 2015; US National Library of Medicine, 2015–2018), and plasma infusion did not provide significant therapeutic benefits (VandeVrede et al., 2020).

One of the kinases associated with increased levels of tau deposits, not only in progressive supranuclear palsy but also in corticobasal degeneration and Alzheimer's disease, is GSK-3 (Ferrer et al., 2002). As mentioned before, GSK-3 is a serine/threonine kinase involved in glycogen synthesis that is important in regulating gene expression and cell survival (Grimes and Jope, 2001). GSK-3 inhibition leads to increased stem cell propagation and neuronal differentiation (Morales-Garcia et al., 2012). Mice with reduced GSK-3β expression show altered synapse condition and lower tau accumulation (Amaral et al., 2021). Another study conducted on mice demonstrated that blocking GSK-3 signaling with tideglusib (thiadiazolidinone) decreases tau phosphorylation and prevents memory deficits in an AD mouse model (Serenó et al., 2009).

Tideglusib (NP031112, NP12) is a GSK-3β inhibitor registered as an orphan drug by the Food and Drug Administration (FDA) and European Medicines Agency (EMA) (Medina, 2018). Tideglusib use in a clinical trial showed no significant changes between drug and placebo in both primary and secondary outcomes (US National Library of Medicine, 2010–2012; Tolosa et al., 2014). According to the anti-inflammatory and neuroprotective activity of tideglusib (Wang et al., 2016), the drug is under investigation in patients with type 1 congenital and childhood-onset myotonic dystrophy (Horrigan et al., 2020). Administration of lithium, which is similar to tideglusib in its mechanism of action, has also been considered as a way to inhibit PSP progression (Engel et al., 2006; Boxer et al., 2017), but the trial was terminated because of adverse effects, including a balance disorder, tremor, fatigue, and urinary tract infections (US National Library of Medicine, 2008–2015).

Studies have also considered indirect mechanisms of GSK-3 inactivation that may lead to stem cell proliferation (Nedachi et al., 2011). Progranulin (PGRN), a growth factor induced by estrogens, triggers GSK3β-phosphorylation and leads to GSK3β inhibition. This results in partial proliferation of neural progenitor cells (NPCs) dependent on GSK inhibition (Nedachi et al., 2011). A trial focused on assessing the correlation between estrogen exposition and PSP occurrence showed no association (Park et al., 2018). On the other hand, the level of PGRN and its cofactor prosaposin has been reported as increased in patients with AD (Mendsaikhan et al., 2019). Using PGRN enhancers as modern targets in neurodegeneration therapies requires further investigation, since its role in pathological processes is not fully understood.

AZP2006, a drug currently in phase 2 clinical trials, is a small, multifunctional molecule that stabilizes the progranulin-prosaposin complex and increases progranulin concentration [ALZFORUM. (n.d.)]. It also inhibits tau phosphorylation and acts to clear misfolded proteins by inducing macroautophagy (Medina, 2018). In 2015, AZP2006 underwent a phase 1 clinical trial, which demonstrated the tolerance of a short 10-day AZP2006 therapy in healthy volunteers (males aged 18–55) [Alzprotect (n.d.)]. Currently, phase 2 of this trial is registered, and participants are being recruited. The goal of the investigation is to evaluate the safety, tolerability, and pharmacokinetics of AZP2006. Pharmacokinetic parameters will be measured in CSF, plasma, and blood in a group of 36 participants either with a placebo or different doses of AZP2006. The drug/placebo will be administered once daily for 84 days. The estimated study completion is planned to be 30 June, 2021 (US National Library of Medicine, 2019a).

Another potential therapeutic progressive supranuclear palsy target is the Src family kinase member Fyn. This non-tyrosine receptor kinase is associated with the regulation of the inflammatory processes, neuronal development, and cancer development (Schenone et al., 2011). In a mouse model, pseudophosphorylation of Fyn was related to an increased level of tau aggregates (Briner et al., 2019). However, a phase 2a trial investigating the safety and tolerability of the Fyn inhibitor Saracatinib (AZD0530) showed little effect on patients with AD, while also revealing a group of adverse effects, mostly affecting the gastrointestinal tract, such as diarrhea and acute diverticulitis (US National Library of Medicine, 2014–2019; van Dyck et al., 2019).

Methylthioninum chloride (methylene blue [MB], TRx0014) is a phenothiazine with known anti-tau accumulation activity used in both *in vitro* (Wischik et al., 1996) and *in vivo* (Melis et al., 2015) studies. MB crosses the BBB and alters cell metabolism *via* the redox process. MB activated the expression of (Nrf2)/ARE genes, and, consequently, reduced inflammatory factors, oxidative stress, and tau accumulation in an animal tauopathy model (Stack et al., 2014). By providing a mammalian target of rapamycin (mTOR) inhibition, MB contributes to the activation of protein phosphatase 2A (PP2A) acting as a GSK-3β antagonist (Meske et al., 2008; Kitagishi et al., 2014; Xie et al., 2014). A study on MB in patients with AD showed a significant improvement in tests evaluating cognitive impairment after 24 weeks of medium MB dose administration (138 mg/day) (US National Library of Medicine, 20007–2008; Wischik et al., 2015).

### Enhancement of Abnormal Protein Removal

Nilotinib (AMN107) is an oral drug classified as a c-Abl tyrosine kinase inhibitor group, primarily used in Philadelphia chromosome-positive chronic myeloid leukemia (CML) treatment (Kantarjian et al., 2007; Ursan et al., 2015; National Cancer Institute, 2020). As a molecule with BBB-crossing ability and the potential to modify autophagy, nilotinib was investigated in animal and human studies (Medina, 2018; Shoeibi et al., 2018). It has been mainly studied in the context of PD and MSA and failed to show a significant benefit (Lopez-Cuina et al., 2020; Pagan et al., 2021). In PD, the c-Abl tyrosine kinase inhibitor reduced α-synuclein levels and induced lysosomal activity (Hebron et al., 2013), whereas in a PSP mouse model, nilotinib decreased phosphorylated tau concentration and improved motor symptoms (Torres-Yaghi et al., 2017), suggesting a potential application in the treatment of tau pathology. However, a phase 2 study analyzing the safety of nilotinib in patients with AD by 6 months of 150–300 mg nilotinib treatment did not produce clinically significant results (Simuni et al., 2021).

The second potential therapeutic approach in tauopathies is the activation of proteasomes. Inhibitors of phosphodiesterases (iPDEs) have proven to be neuroprotective (Kumar and Khanna, 2017). By increasing cellular cAMP level, providing cAMP response element binding (CREB), and activating cAMP-dependent protein kinase A (PKA), iPDEs lead to improvement in memory consolidation and enhanced degradation of misfolded proteins *via* proteasomal activation (García-Osta et al., 2012; Lokireddy et al., 2015). CREB is a transcription factor that controls the expression of genes connected with neuroplasticity and neuroprotection; CREB modification can lead to a significant cognitive improvement (Sakamoto et al., 2011). Targeting the cAMP/PKA/CREB pathway brought significant results in other neurodegeneration therapies, such as those for the treatment of AD (Gong et al., 2004). cAMP activates the proteasome, leads to tau removal, and modifies disease progression in tauopathies such as PSP. Furthermore, reduced activity of AMP-activated protein kinase (AMPK) in a PS19 mouse model led to reductions in phosphorylated tau deposits (Domise et al., 2016).

Recent trials have shown other encouraging results, indicating, apart from the decreased accumulation of misfolded tau protein, anti-inflammatory effects after the application of rolipram, a selective PDE-4 inhibitor (Zhu et al., 2001; Gong et al., 2004). Another human study on healthy male volunteers showed that the administration of 100 mg of sildenafil, a PDE-5 inhibitor, resulted in improvement of cognitive functions. Despite this, further investigation is needed to evaluate the activity of sildenafil in the CNS (Schultheiss et al., 2001). AZP2006, which stabilizes prosaposin-progranulin complexes, is also included in a group of medicaments triggering tau degradation. It was described in the previous section [Shoeibi et al., 2018, ALZFORUM. (n.d.)].

### Microtubule Stabilization

There are also drugs suspected to stabilize microtubules via specific mechanisms. This group includes neomycin (aminoglicoside antibiotic regulating exon 10 splicing but not demonstrating enough selectivity) (Varani et al., 2000) and mitoxantron (acting also as a stem-loop stabilizer) (Zheng et al., 2009). Despite a promising mechanistic connection, neither of these compounds has found its clinical application in PSP treatment. Davunetide, which produced microtubule stabilization and reduced tau phosphorylation in preclinical studies, has also failed in clinical trials examining use for PSP treatment (Boxer et al., 2014).

TPI-287, part of the taxoid family, is a tubule-binding and microtubule-stabilizing molecule. The clinical trial, which was conducted simultaneously with a group of patients with AD, investigated the safety and tolerability of TPI-287 administration in patients with primary four repeat tauopathies (4RT), PSP, and CBS by the intravenous infusion of TPI-287 in three cohorts (Sakamoto et al., 2011; García-Osta et al., 2012). Each patient received an increasing dose of TPI-287 (2–6, 3–20 mg/m^2^ depending on the trial arm), which was preceded by premedication that included diphenhydramine, dexamethasone, and famotidine. The results were compared with those of the fourth group that was given a placebo (US National Library of Medicine, 2014–2020). Although TPI-287 was not detectable in the CSF, the trial showed decreased chitinase-3-like protein-1 (YKL-40) levels in the 4RT arm, which suggested a reduction in inflammation due to TPI-287 administration. In the 4RT-arm, there were also dose-dependent cognitive impairments and an increased amount of falls reported (Tsai et al., 2020).

Another compound demonstrating a similar mechanism of action is epothilone D (BMS-241027), a microtubule stabilizing agent with the ability to pass through the BBB (US National Library of Medicine, 2014–2020). Studies on mice showed that low epothilone D doses reduce the amount of cerebral pathologic tau and inhibit axonal destruction, concomitantly contributing to improved axonal transport (Barten et al., 2012; Zhang et al., 2012a; Makani et al., 2016; Clark et al., 2020). Trial with patients with AD by weekly epothilone D infusions (0.003, 0.01, and 0.03 mg/kg doses) evaluated safety and pharmacodynamic properties, and included a measure of CSF drug concentration [US National Library of Medicine, 2011–2014, ALZFORUM. (n.d.)]. Although the trial was finished in October 2013, no data were reported because of trial termination [ALZFORUM. (n.d.)].

Dictyostatin is also considered as a potential option in future tauopathy treatment. Dictyostatin is a macrolide with microtubule-stabilizing and anticancer properties (Paterson et al., 2010), and acts like epothilone D (Makani et al., 2016). High-dose intraperitoneal injection of dictyostatin in mice led to significant body weight loss, gastrointestinal overgrowth, and death. Low-dose infusions, although also induced some deaths, resulted in the reduction of both atrophy of hippocampal CA3 neurons and levels of mature tau markers (Makani et al., 2016). There are no ongoing clinical trials with dictyostatin. Studies using discodermolide, an anticancer drug with great similarities to dictyostatin, did not show improvement, despite initially promising results. Moreover, discodermolide showed high toxicity that led to pulmonary interstitial disease (Mita et al., 2004; Falkenberg et al., 2019; Guo et al., 2020).

### Stem Cell Administration

Since progressive supranuclear palsy is not only considered as a disease of the central nervous system, it seems crucial to explore cells that are not strictly connected with the nervous system. For that purpose, studies have examined mesenchymal stromal cells (MSCs) obtained from patients with PSP (Calogero et al., 2018). The results showed deficient microtubule polymerization and depolarization processes, which led to their inefficient remodeling. The proliferative capacity of MSCs was also greatly decreased in comparison with the control group. This served as the first evidence that disruption in microtubules stability is present not only in the nervous system. *In vitro* investigations proved that the ROCK and PI3K pathways are strictly involved in the MSCs transport within the BBB (Lin et al., 2013).

Mesenchymal stromal cells can differentiate into cells of every germinal layer: ecto-, endo-, and mesoderm, whereas brain-derived neurotrophic factor (BDNF) and retinoic acid (RA) induce the transformation of MSCs into neuronal cells (Anghileri et al., 2008). A significant feature of MSCs for therapeutic use in PSP is their ability to decrease oxidative stress level and influence the apoptotic process. It has been established that MSCs of PSP patients can penetrate the BBB and produce neurotrophins in the same amount as MSCs obtained from healthy participants (Giordano et al., 2014). Neurotrophins are factors with a potential for controlling almost every important process in the cell, including both its survival and apoptosis (Chao et al., 2006). Factors belonging to this group, such as BDNF, are crucial for the sustainability of daily brain activity (Houlton et al., 2019).

Studies in a transgenic Alzheimer's disease mouse model showed that the injection of human adipose derived stem cells (hASCs) significantly increased the level of anti-inflammatory interleukin-10, suggesting the neuroprotective effect of hASCs. This manipulation also increased the concentration of the postsynaptic density protein-95 (PSD-95) as well as synaptophysin, markers indicating synaptic and dendritic stabilization (Kim et al., 2012). hASCs have also been effective against 6-hydroxydopamine (6-OHDA)-induced damage (Cova et al., 2012).

Stem cells are an active area of clinical research, and there have been several PSP clinical trials with promising results. Those studies demonstrate the safety of intraarterial MSC administration in patients with PSP and suggest that while clinical stabilization is possible, it may be disappointingly temporary compared with expectations (US National Library of Medicine, 2013; Giordano et al., 2014; Canesi et al., 2016). However, there are still no data available to definitively confirm the efficacy of this novel therapeutic approach. Relatively small population and short follow-up period are the main limitations of stem cell-based therapy for PSP. This area requires further research in order to unequivocally prove the advantages of this approach.

Another origin of mesenchymal stromal cells could be adipose tissue; the first case of a therapy using this cell source was described in South Korea in 2014 (Choi et al., 2014). A patient diagnosed with PSP at the age of 71 demonstrated no response to benserazide and levodopa treatment; so, in order to overcome pharmacological resistance, MSCs were taken by liposuction from the subcutaneous tissue of the patient and administered by five intravenous infusions by the cephalic vein and four intrathecal depositions. There were no serious adverse effects reported, excluding recurrent mild fever and a one-time blood pressure increase, which were relieved after an antipyretic medicament. Safety assessment and clinical progress were provided regularly during the 6-month period. A short follow-up demonstrated a significant reduction (from 69 to 63 points) in PSPRS (Golbe and Ohman-Strickland, 2007; Choi et al., 2014). The strength and speed of activities performed with upper limbs also improved. Although this kind of treatment was only performed on one patient and with a short follow-up time, this form of therapy may hold promise in the future.

A future study analyzing stem cell administration is currently recruiting participants. It plans to transfer bone marrow stem cells (BMSCs) to the vascular system and to the nasal cavity, with the premise that the branches of the trigeminal nerve located in conches and meatuses will allow stem cells to enter the CSF (US National Library of Medicine, 2016). The assessment is based on Activity of Daily Living Scale evaluation 3, 6, and 12 months after administration. The trial is planned to be finished by June 2023 (US National Library of Medicine, 2016; Edemekong et al., 2020).

### Specific Antibodies

There is an increasing interest in developing molecule-specific antibodies, since neutralization of tau aggregates seems to be a crucial step in progressive supranuclear palsy treatment, and novel trials are suspected to bring positive results. On one hand, the intracellular nature of tau limited the pursuit of immunotherapy usage in patients with tau pathologies (Avila, 2010; Agadjanyan et al., 2017). On the other hand, the discovery of tau axonal propagation and its ability to transmit into other unaffected neurons challenged this theory, concomitantly providing the argument for constructing monoclonal antibodies (Clavaguera et al., 2009). Both passive (Chai et al., 2011) and some active forms of immunization were assessed as effective (Theunis et al., 2013).

The liposome-based amyloid vaccine (ACI-35) has had a significant effect on a P301L tau transgenic mouse mode, not only in short-term evaluation by the sudden expression of specific tau-antibodies but also in general improvement of motor impairment in comparison with the control group (US National Library of Medicine, 2020f). Studies on humans have also brought positive results, although the primary version of the vaccine was too weak to induce an immunological response (ALZFORUM, 2020b, 2021; US National Library of Medicine, 2020f). ACI-35.030, the second version of the ACI-35 vaccine containing another adjuvant, provides stronger T helper cell activation and leads to 50 times more intense antibody production (ALZFORUM, 2020b, 2021). Infusion of increasing vaccine doses caused a significant change in IgG-antibody titers and did not cause adverse effects (AC Immune, 2021). The last stage of the trial, evaluating the effect of the highest vaccine dose, is planned to be finished by 2023 (ALZFORUM, 2021).

Armanezumab is a monoclonal, humanized antibody that targets the N-terminal region of the tau protein, called the “phosphatase activation domain”. When tau is pathologically accumulated, its N-terminal region, normally hidden in a “paperclip” shape, becomes visible and plays a significant role in tau propagation. Studies on tau transgenic mice demonstrated the therapeutic activity of armanezumab, shown by decreased levels of tau spread, aggregation, and toxicity with simultaneously high specificity (Agadjanyan et al., 2017).

AV-1980D is an anti-tau deoxyribonucleic acid vaccine acting against the same epitope as armanezumab (Davtyan et al., 2017; Shoeibi et al., 2018). A study using a THY-Tau22 mouse model demonstrated a strong humoral reaction, resulting in the production of antibodies. Nevertheless, this powerful reaction did not show any harmful adverse effects and, while the pool of antibodies was restricted, its level remained stable (Davtyan et al., 2017). *In vivo* trials of AV-1959R and AV-1980R, vaccines created against Aβ and tau proteins, also demonstrated high affinity of antibodies and significant neutralization of tau aggregates (Davtyan et al., 2019). A human study on the safety and efficacy of anti-tau antibody infusion was announced in 2019. Fifty-two healthy male adults participated in phase 1 of a bepranemab (UCB0107) intake trial, and all of them completed the study. None of the adverse effects of the trial were serious, the most common being a headache. UCB0107 became the next drug candidate targeting the underlying cause of PSP and still remains a subject of subsequent studies (UCB, 2019; ALZFORUM, 2020c).

AADvac1 is a form of an active vaccine, the production of which was based on the specific fragment causing tau oligomerization (Shoeibi et al., 2018). *In vivo* trials conducted on a transgenic AD rat model showed the safety profile of AADvac1 and its positive influence on clinical disease symptoms (Kontsekova et al., 2014). Encouraging results of animal-based studies led to human trials investigating AADvac1 efficacy in AD (US National Library of Medicine, 2013–2015, 2014–2017a, 2015–2019) and primary progressive aphasia (US National Library of Medicine, 2017). Recently published results of phase 2 of the AADvac1 in patients with mild Alzheimer's disease (ADAMANT) trial demonstrated that AADvac1 is safe and effective. The production of specific antibodies suggests the development of an accurate immune response (Axon Neuroscience, 2019), providing evidence for potential AADvac1 efficacy. Finally, these specific antibodies were reported to form peculiar complexes with tau aggregates in tau-affected human neurons (ALZFORUM, 2020a).

Antibodies against the microtubule binding domain (MTBD) may also be potentially useful in the treatment of progressive supranuclear palsy. Recently published data show the effectiveness of injections of such antibodies. These results showed a high affinity for tau and responses against tau aggregates in a mouse model (Croft et al., 2018). Additionally, two other anti-tau antibodies, tilavonemab (ABBV-8E12) (US National Library of Medicine, 2016–2021) and gosuranemab (BIIB092), were shown to be ineffective as PSP treatments (US National Library of Medicine, 2017–2020). Nevertheless, one of them was also suspected to have a therapeutic potential in other forms of neurodegeneration (US National Library of Medicine, 2018–2019).

The BIIB092 investigation in progressive supranuclear palsy (PASSPORT) was evaluated in a TauBasket trial, which included patients with corticobasal degeneration, frontotemporal dementia (FTLD), traumatic encephalopathy syndrome (TES), symptomatic MAPT (sMAPT) mutation carriers, and progressive nonfluent aphasia (ALZFORUM, 2019). One-hour intravenous infusion every 4 weeks for a 20-week period was administered to assess safety and tolerability, primarily measuring adverse events caused by BIIB092 in comparison with placebo administration (US National Library of Medicine, 2018–2019). The study completion date was originally estimated to be December 19, 2019, but the trial was suspended earlier than scheduled because the primary end point was not met (US National Library of Medicine, 2017–2020; ALZFORUM, 2019).

### Corticobasal Degeneration

Corticobasal degeneration is a form of atypical parkinsonism histopathologically characterized by aggregates of tau protein with four microtubule-binding repeats similar to those observed in progressive supranuclear palsy (Reich and Grill, 2009; Chahine et al., 2014). Because of a similar underlying pathology, novel therapeutic approaches for both CBD and PSP can be discussed together. Additionally, several other clinical phenotypes related to CBD can be distinguished: corticobasal syndrome (CBS), nonfluent/agrammatic variant of a primary progressive aphasia (naPPA), frontal behavioral-spatial syndrome (FBS), and progressive supranuclear palsy-like syndrome (PSPS) (Armstrong et al., 2013). Clinically, the diagnosis of CBD is now referred to CBS, which can be a manifestation of pathologies beyond CBD, as it overlaps with several forms of dementia of differing neuropathologies (Armstrong et al., 2013). As a result of its diverse clinical manifestations and a lack of biomarkers enabling early diagnosis, it is estimated that only 25-56% of patients are diagnosed correctly with CBS before death (Lee et al., 2011; Alster et al., 2018, 2020; Svenningsson, 2019; Caixeta et al., 2020).

The causes of corticobasal degeneration are currently unclear. The H1-haplotype of the MAPT gene expressed in CBD and PSP is one of them (Houlden et al., 2001; Chahine et al., 2014). In several CBS cases, a specific mutation (p.V363I) in the MAPT gene has also been identified (Ahmed et al., 2019). The clinical features of CBS, PSP, and frontotemporal dementia (FTD) are included in the FTLD spectrum (Armstrong et al., 2013). It is worth mentioning that FTLD more often manifests with inclusions containing ubiquitin, not tau protein as seen in PSP. Mutations in the progranulin gene (PGRN), already described in FTLD, have also been discovered in patients with the familial occurrence of CBS (Masellis et al., 2006). By affecting different areas of the human brain, tau aggregation can manifest not only by the presence of involuntary activities, such as myoclonus and rigidity, but also as cognitive impairment (Chahine et al., 2014). Asymmetry is a characteristic element of the physical examination for CBD, which is a helpful criterion for diagnosis (Reich and Grill, 2009). Typical histopathological changes in CBS manifest as a sparse composition of tiny filaments localized to the cerebrum (Foltynie and Athauda, 2018). Therefore, it is preferred to diagnose CBS based on its clinical features, and a definitive CBD diagnosis should be reserved for postmortem histological confirmation (Reich and Grill, 2009).

The prevalence of progressive supranuclear palsy, corticobasal syndrome, and frontotemporal dementia is estimated to be 10.8/100,000. Their incidence index does not diverge remarkably from mortality (Coyle-Gilchrist et al., 2016). Although studies comparing CBD occurrence between sexes have been ambiguous, most have shown equal frequency (Dickson, 1999). Unfortunately, no specific treatment against CBD exists [National Organization for Rare Disorders. (n.d.)]. Because of the modest level of CBD incidence and non-specific symptoms, only a small group of clinical trials have been conducted in CBD. These trials include Fasudil (ROCK-inhibitor) (US National Library of Medicine, 2021), TPI-287 (microtubule-stabilizing molecule) (US National Library of Medicine, 2014–2020; Tsai et al., 2020), and gosuranemab (anti-tau antibody) (US National Library of Medicine, 2018–2019), mentioned in the PSP section. Current CBD treatment is primarily based on relieving its symptoms by pharmacotherapy (Caixeta et al., 2020). Potential novel therapeutic approaches for PSP and CBS are summarized in Table 1.

**Table 1:** Progressive supranuclear palsy (PSP) and corticobasal syndrome (CBS): Summary of emerging novel therapies.

**Type of action**	**Drug**	**Phase**	**Status**	**Outcome/end points**	**References**
Inflammation modulation	5-lipooxygenase (5-LO)	Pre-Clin	Completed	Overexpression of 5-LO gene showed enhanced amyloid-β (Aβ) aggregation	Boxer et al., 2017
	5-lipooxygenase (5-LO)	Pre-Clin	Completed	5-LO block led to decreased tau levels and memory improvement	US National Library of Medicine, 2016
	Benfotiamine (BFT)	Pre-Clin	Completed	BFT reduced the level of MAPT and amyloid plaques	Färber and Kettenmann, 2005
	Benfotiamine (BFT)	Pre-Clin	Completed	BFT reduced the level of glycated tau and improved mice behavior	Maphis et al., 2015
	Benfotiamine (BFT)	Pre-Clin	Completed	BFT considered as a anti-inflammatory and neuroprotective factor	Chu et al., 2012
	Tolfenamic acid (TA)	Pre-Clin	Completed	TA reduces the total tau-distributionin mice central nervous systems	Marras et al., 2018
	Tolfenamic acid (TA)	IIa	Not yet recruiting	Safety and tolerance of tolfenamic acid in individuals with PSP; CSF evaluation	Tapias et al., 2018
Modulation of oxidative stress	PERK activation	Pre-Clin	Completed	Reduction of 4R-tau level and tau phosphorylation	Chirichigno et al., 2002
	PERK activation	Pre-Clin	Completed	Improvement of motor and cognitive functions, decreased tau phosphorylation	Chirichigno et al., 2002
	SLC25A38/Appoptosin regulation	Pre-Clin	Completed	Increased level of Appoptosin is connected with tau clevage and motor functions impairment	Zhao et al., 2015
	Coenzyme Q10	II	Completed	Gentle improvement of PSP symptoms measured by PSPRS and FAB	Stamelou et al., 2008
	Coenzyme Q10	Clinical	Completed	Primary outcome measures: efficacy of Coenzyme Q10 UPDRS and PSPRS; no significant results	Apetauerova et al., 2016
	Creatine, pyruvate, niacinamide	Clinical	Completed	Primary outcome measures: clinical features of PSP, including motor function, neuropsychological function, and blood chemistry; results were not published	US National Library of Medicine, 2019c
	α-lipoic acid and L-acetyl carnitine	Pre-Clin	Completed	α-lipoic acid and L-acetyl carnitine have a neuroprotective effect	DeVos et al., 2017
	α-lipoic acid and L-acetyl carnitine	II	Completed	Primary outcome measures: incidence and severity of adverse events: the most common adverse effects were: restlessness, seizures, insomnia and dizziness	DeVos et al., 2017
	-lipoic acid and L-acetyl carnitine	Pre-Clin	Completed	ALCAR led to induction of mitochondrial restoration and was convinced to present anti-oxidative properties	DeVos et al., 2013
	NBMI - N,N'-bis (2-mercaptoethyl) isophthalamide	IIa	Recruiting	Primary goal is to evaluate NMBI influence on motor, non-motor syndromes and check the QoL index in PSP and MSA patients	MacDonald et al., 2009
RNA modulation	ASO	Pre-Clin	Completed	ASO reduced tau mRNA and protein in the brain, spinal cord, and CSF	Ferrer et al., 2001
	ASO	Pre-Clin	Completed	ASO reduced the severity of seizures observed in these models	Iqbal et al., 2008
	ASO	I	Recruiting	Primary outcome measures: number of adverse effects, change in severity scores for C-SSRS and levels of infection indicators in CSF	Bruch et al., 2015
	siRNA	*In vitro*	Completed	siRNA leads to GSK3α and GSK3βinhibition	Yuan et al., 2018
Kinases and enzymes modulation	ROCK inhibition	II	Recruiting	Primary outcome measures: number of adverse effects assessed in psychical examination, imaging and laboratory tests	Bruch et al., 2017
	Pin1 inhibition	Pre-Clin	Completed	Inhibition of cis-trans cover; antibodies used against cis-form prevented from tauopathy development	Riento and Ridley, 2003
	CDK5/BAG3/Hsp70 path targeting	*In vitro*/Pre-Clin	Completed	BAG3 loss resulted in loss of memory functions and disruption of neuronal homeostasis	Silva and Haggarty, 2020
	O-GlcNAcase inhibitor	Pre-Clin	Completed	*O*-GlcNAc modification decreases tau aggregation and leads to decreased level of neuronal loss	Wells et al., 2002
	ASN120290 (O-GlcNAcase inhibition)	I	Completed	Drug remained safe and well-tolerated	Yuzwa et al., 2012
	ASN120290 (O-GlcNAcase inhibition)	I	Recruiting?	Main goal of trial is to calculate OGlcNAcase enzyme occupancy by ASN120290 in CSF	Chen et al., 2018
	MK-8719	Pre-Clin	Completed	Reduction of tau aggregates and diminishment of forebrain atrophy	Hastings et al., 2017
	MK-8719	I	Completed	Drug was well-tolerated and its level was proportional with the dose	ALZFORUM. (n.d.)
	GSK-3β hemi-knockout	Pre-Clin	Completed	Decrease of GSK-3β leads to inhibition of tau phosphorylation and aggregation	Ferrer et al., 2002
	Tideglusib (NP031112, NP12)	Clinical	Completed	Tideglusib was acclaimed as safe, although trial showed also no significant changes between drug intake or placebo both in primary and secondary outcomes	Serenó et al., 2009; Amaral et al., 2021
	AZP2006	II	Recruiting	Primary outcome measures: tolerability, safety, pharmacokinetics and effect of AZP2006; it also includes CSF markers evaluation	Mendsaikhan et al., 2019
	Methylene blue, TRx0014	Pre-Clin	Completed	MB reduced tau pathology and inflammation, it also showed improvement in mice behavior	van Dyck et al., 2019
	Methylene blue, TRx0014	II	Completed	Benefit was seen on the ADAS-cog scale in both mild and moderate subjects	van Dyck et al., 2019
Enhancement of abnormal proteins removal	Nilotinib (AMN107)	Pre-Clin	Completed	Treatment led to decreased level of tau aggregates and improvement in motor symptoms	National Cancer Institute, 2020
	Rolipram (PDEs inhibitor)	*In vitro*/PreClin	Completed	Rolipram is suspected to decrease level of inflammation in CNS, although it has different biological effects	Lokireddy et al., 2015
	Sildenafil (PDE-5 inhibitor)	I	Completed	Enhanced ability to focus attention and select relevant target stimuli in the sildenafil condition; further studies are being required	García-Osta et al., 2012
Microtubules stabilization	TPI-287	I	Completed	Trial showed decreased chitinase-3-like protein-1 (YKL-40) levels in the 4RT arm, what presumably evidenced reduction of inflammation due to TPI-287 administration	Schultheiss et al., 2001; Zhu et al., 2001
	Epothilone D (BMS-241027)	PreClin	Completed	Drug reduces the amount of cerebral pathologic tau and also inhibits axonal destruction, concomitantly contributing to the axonal transport improvement	Zheng et al., 2009
	Dictyostatin	Pre-Clin	Completed	Low doses infusion showed decrease of hippocampal CA3-neurons atrophy and lowered the level of mature-tau markers	Zheng et al., 2009
Stem cells administration	hASCs	Pre-Clin	Completed	hASCs significantly decrease the level of inflammation and indicates synaptic and dendritic stabilization	Lin et al., 2013
	BMSCs	II	Completed	Almost all treated patients were alive after one year cell infusion, the motor function rating scales remained stable for at least six-months	Chao et al., 2006; Giordano et al., 2014
	AdMSCs	Human study performed on one patient in South Korea using MSCs obtained from adipose tissue	Completed	No serious adverse effects, significant reduction in PSPRS, strength and speed of activities performed with upper limbs have also improved	Schneider and Mandelkow, 2008
	BMSCs	Clinical	Recruiting	Primary outcome measures: Activities of Daily Living (ADL)	Höglinger et al., 2017
Specific antibodies	ACI-35 vaccine	Pre-Clin	Completed	Sudden expression of specific tau-antibodies, general improvement of motor impairment compared to the placebo group	Edemekong et al., 2020
	ACI-35.030 vaccine	Clinical	Last stage of trial, evaluating effect of the highest vaccine dose is planned to be finished in 2023	Significant change in IgG-antibodies titers and did not cause adverse effects	Clavaguera et al., 2009
	Armanezumab	Pre-Clin	Completed	Decreased level of tau scattering, aggregation and toxicity with simultaneously high specificity	Canesi et al., 2016
	AV-1980D	Pre-Clin	Completed	Strong humoral reaction resulting in antibodies production	Theunis et al., 2013
	AV-1959R and AV-1980R	Pre-Clin	Completed	High affinity of antibodies and significant reduction of tau aggregates	US National Library of Medicine, 2020f
	Bepranemab (UCB0107)	I	Completed	All participants had completed the study, there were no serious adverse effects	ALZFORUM, 2021
	AADvac1	Pre-Clin	Completed	Proved AADvac1 safety profile and exhibited its positive influence on clinical disease symptoms	AC Immune, 2021
	AADvac1	II	Completed	AADvac1 is safe and presents accurate immunogenicity	UCB, 2019
	Antibodies against microtubule binding domain (MTBD)	Pre-Clin	Completed	All of the new tau antibodies detected human tau in whole brain lysates from PS19 mice	US National Library of Medicine, 2014–2017a
	BIIB092 (gosuranemab)	I	Terminated	Primary outcome measures: incidence of Treatment-Emergent Adverse Events	Axon Neuroscience, 2019

## Multiple System Atrophy

Multiple system atrophy is a fatal neurodegenerative disease characterized by rapid progression and low life expectancy. Mean survival rate is estimated to be 6–10 years (Monzio Compagnoni and Di Fonzo, 2019). Until recently, multiple system atrophy (MSA) was considered a sporadic disease, but some cases have been observed among families, suggesting a potential genetic predisposition (Gilman et al., 2008; Jellinger and Lantos, 2010; Jellinger, 2018; Palma et al., 2018). MSA symptomatology consists of classic parkinsonian motor symptoms accompanied by dysautonomia and cerebellar ataxia. The onset of autonomic symptoms occurs earlier in comparison with that of Parkinson's disease. Depending on the clinical manifestation, there are two main subtypes distinguished: MSA-P and MSA-C. MSA-P, which is diagnosed in 80% of all cases in Europe, is characterized by predominantly parkinsonian motor symptoms. In MSA-C, cerebellar symptoms such as gait ataxia, dysarthria, oculomotor dysfunction, and intention tremor are predominant (Jellinger, 2018). Rarely, the MSA-A subtype will present with autonomic failure consisting of orthostatic hypotension, urinary incontinence, erectile dysfunction, and constipation. According to the current consensus criteria for the diagnosis of MSA, a definite diagnosis can only be made through postmortem examination of the brain showing α-synuclein cytoplasmic inclusions in oligodendroglia cells and neurons (Gilman et al., 2008). Based only on the clinical manifestation, a possible or probable diagnosis can be made.

Multiple system atrophy, as one of the synucleinopathies, is characterized by pathological accumulation and aggregation of α-synuclein mainly in the cytoplasm of oligodendroglia cells (Monzio Compagnoni and Di Fonzo, 2019). These so called “glial cytoplasmic inclusions” (GCIs) consist mainly of aggregates and some other proteins such as ubiquitin, tau protein, p62, and heat shock protein (Jellinger and Lantos, 2010). α-Synuclein is natively unfolded but is considered to aggregate and alter because of oxidative stress, mutations, decrease in neurotrophic factors, and inflammation (Monzio Compagnoni and Di Fonzo, 2019). Mitochondrial dysfunctions, especially mitochondrial-based mutations, have also been shown to influence the pathogenesis of neurodegenerative diseases (Schapira, 2008). Thus, targeting those causes remains a reasonable approach for developing novel therapies for MSA and other synucleinopathies. Since the exact pathogenesis of MSA remains unclear, current therapeutic approaches are again limited to controlling clinical symptoms and improving quality of life.

### α-Synuclein

α-Synuclein, a product of the alpha-synuclein (SNCA) gene (Kisos et al., 2012; Watts et al., 2013; Djelloul et al., 2015; Burré et al., 2018; Coon and Singer, 2020), is an aggregation-prone protein that plays an important role in several synucleinopathies (Surguchev and Surguchov, 2017). Since α-synuclein aggregates in oligodendroglia, a hallmark of MSA, and the presence of misfolded, pathological accumulation of this protein are a main culprit for developing MSA symptoms, α-synuclein is a primary target for potential MSA therapies. Natively, α-synuclein is present as a soluble, unfolded protein. α-Synuclein aggregation leads to both suppression of neurotrophic factors secretion and neuronal loss, especially in the olivopontocerebellar and nigrostriatal regions (Jellinger, 2018). Although its exact function remains unclear, α-synuclein appears to play a role in neurotransmitter release and synaptic plasticity. Physiologically, α-synuclein is produced in neurons and is located in presynaptic neuronal terminals (Watts et al., 2013). Although it is produced by neurons, the mechanism of transfer of α-synuclein to oligodendroglia cells remains undefined. There is a hypothesis confirmed by preclinical trials showing cell-to-cell transmission from neurons to oligodendrocytes through synapses, but the exact origin of GCIs is still unclear (Kisos et al., 2012; Watts et al., 2013; Burré, 2015; Djelloul et al., 2015; Brundin et al., 2017; Burré et al., 2018; Coon and Singer, 2020).

#### Neutralizing α-Synuclein Aggregation

Studies with an MSA mouse model showed Anle 138b, an oral general inhibitor of protein aggregation, to be successful in reducing oligomeric α-synuclein concentration and glial cytoplasmic inclusions. This leads to improvement of motor function in Anle 138b-treated mice compared with controls (Wagner et al., 2013; Heras-Garvin et al., 2019). This appears to be a promising approach to suppress MSA and other neurodegenerative diseases progress. Thus, a phase 1 clinical trial assessing the safety and tolerance of oral administration of Anle 138ba among patients with Parkinson's disease has been initiated recently (US National Library of Medicine, 2020e).

The mammalian target of rapamycin kinase signaling pathway is widely known for its role in controlling cell metabolism and stimulating cell proliferation. It is also a suppressor of catabolic pathways and a promotor of anabolic ones, such as protein and lipid synthesis (Kim and Guan, 2015). It has been shown in preclinical trials that rapamycin, an mTOR inhibitor, increases autophagy and, therefore, removal of abnormal proteins, such as α-synuclein, inhibiting its accumulation (Lopez-Cuina et al., 2018; Gao et al., 2019). Oral sirolimus, an mTOR inhibitor, has been examined as a possible treatment to inhibit the neurodegenerative process in MSA. Recently, a new randomized placebo-controlled 2 phase trial assessing the efficacy of oral sirolimus in suppressing MSA progression began (US National Library of Medicine, 2018); however, the study was terminated because of early evidence of sirolimus futility. Further research considering modulation of the mTOR kinase signaling pathway as a possible approach in APS treatment is required.

The administration of epigallocatechin gallate (EGCG), a polyphenol found in green tea, was also evaluated as a possible anti-aggregation approach. Preclinical trials with mouse models showed that EGCG inhibited α-synuclein aggregation and reduced its toxicity. Unfortunately, the phase 3 clinical placebo-controlled trial showed no improvement in disease progression (Levin et al., 2019).

Several studies have shown that impairment of the ubiquitin-proteasome system (UPS) contributes to the pathogenesis of synucleinopathies such as Parkinson's disease or multiple system atrophy (Tanji et al., 2013). Dysfunction of this protein degradation pathway leads to abnormal aggregation and accumulation of α-synuclein in oligodendroglia and formation of GCIs (Tanji et al., 2013). Studies on mice showed that systemic proteasome suppression (PSI) resulted in increased cytoplasmic accumulation of α-synuclein and reduced motor function in mice with MSA compared with wild-type mice. Neuronal loss in the nigrostriatal and olivopontocerebellar regions of the brain, areas typically associated with MSA neurodegeneration, also occurred in transgenic mice after PSI (Stefanova et al., 2012b). These data suggest that enhancing proteasome activity in patients with MSA could act as a possible disease-modifying treatment. α-Synuclein was shown to integrate with neuron-specific microtubule- β-III tubulin in transgenic mice models, resulting in the formation of insoluble complexes that aggregated in neurons (Nakayama et al., 2009). Furthermore, the same study revealed that microtubule depolymerizing treatment, which decreased α-synuclein and β-III tubulin binding, inhibited α-synuclein accumulation (Nakayama et al., 2009). Thus, microtubule formation-targeting treatment may be another pathway for addressing neurodegeneration in MSA.

#### Immunization

Active immunization, to induce a long-lasting antibody response, may reduce α-synuclein aggregation. Antibodies are believed to increase alpha-synuclein destruction and, therefore, inhibit its aggregation in oligodendrocytes. PD01 and PD03 peptide vaccines consist of short amino acid sequences that are complementary to a segment of α-synuclein. The short peptides are bound to the carrier protein recognized by T-helper cells and help to develop a long-lasting immune response. Studies conducted on transgenic mice have shown that administration of PD01 vaccine, which mimics the C-terminal peptide of α-synuclein, induced a specific immune response against α-synuclein through production of antibodies. This response reduced α-synuclein accumulation in glial cells and decreased demyelination in both the neocortex and striatum. Improved motor skills were also observed in PD01-treated mice (Mandler et al., 2015). Recently, a phase 1 randomized placebo-controlled clinical trial has been completed to assess the safety, tolerance, and immunogenicity of subcutaneous injections of PD01 and PD03 in patients with early MSA. The results of the trial showed the vaccines to be safe and well-tolerated, as well as highly immunogenic, since the treatment with either peptide resulted in considerable IgG antibody production against PD01 and PD03 (US National Library of Medicine, 2014–2017b; Meissner et al., 2020). Although the outcomes appear promising, further studies in this area are needed.

Another possible strategy is passive immunization with specific α-synuclein-targeting antibodies. Preclinical trials showed that injections of anti-α-synuclein antibody in transgenic mice resulted in a decrease in α-synuclein accumulation in spinal cord and hippocampus and lowered GCI concentration in oligodendroglia (Kallab et al., 2018).

### Addressing Neuronal Loss

#### Stem Cell Therapy

Recently, another potential therapeutic pathway for addressing neuronal loss has been developed using autologous mesenchymal stem cells (MSCs) in multiple system atrophy and other parkinsonian syndromes therapy (Shin and Lee, 2020). MSCs derived from human bone marrow and other tissues have the capability to differentiate into various types of cells such as osteoblasts, chondrocytes, fibroblasts, or neurons. MSCs secrete some neuroprotective factors, immunomodulating cytokines, and neurotrophic agents that exert an anti-inflammatory effect, suppress fibrosis and apoptosis, and promote cell differentiation and proliferation. In this way, MSCs act as tissue microenvironment modulators (Caplan and Dennis, 2006). Non-clinical trials with MSA mouse models confirmed that MSCs prevent neuronal loss in striatum and nigrostriatal pathway through the expression of neuroprotective cell survival factors. MSCs are also able to suppress microglia activation and the inflammatory process (Park et al., 2011; Stemberger et al., 2011). A randomized placebo-controlled trial on patients with MSA-C revealed that intravenous/intraarterial MSC administration resulted in slowed disease progression compared with placebo as shown by Unified MSA Rating Scale (UMSARS) scores (Lee et al., 2012). A recently completed phase 1 clinical trial showed that a single intraarterial administration of MSCs in patients with MSA-C was safe and generally well-tolerated (Chung et al., 2020). Currently, ongoing studies aim to collect long-term follow-up data regarding the efficacy and adverse effects of MSC treatments on the patients who took part in the phase 1 trial (US National Library of Medicine, 2020d). Another possible way of administrating MSCs has been recently assessed in a phase 1/2 placebo-controlled clinical trial. Intrathecal administration of adipose-obtained MSC did not cause any serious adverse effects and was also proven to be safe. Disease progression, assessed with UMSARS scores, was slower among patients who received MSC treatment (Singer et al., 2019). All of the above-mentioned trials indicate that mesenchymal autologous cells could be used in the treatment of MSA and are, thus, worth investigating further.

#### Insulin Resistance Targeting

Studies on mouse models and patients with multiple system atrophy showed insulin resistance in oligodendrocytes and neurons of the putamen and impairment of insulin/IGF-1 signaling. These changes may affect the functioning of oligodendrocytes and result in neurodegeneration in the putamen (Bassil et al., 2017). Insulin has been shown to have neuroprotective and neurotrophic effects on neurons (Shemesh et al., 2012). Recently, a double-blind placebo-controlled study assessing the effect of intranasal insulin injection on motor and cognitive skills of patients with PD and MSA has been completed. The results showed that patients who received insulin performed better in cognitive and motor assessments compared with the placebo group (Novak et al., 2019). Nevertheless, further investigation is crucial to assess the safety of intranasal insulin administration and to confirm its positive effects as a potential treatment. Recently, glucagon-like peptides have also been examined as a possible treatment in synucleinopathies (Foltynie and Athauda, 2018). Preclinical studies showed that injection of Exendin-4, a glucagon-like peptide 1 analog, lowered insulin resistance and suppressed neuronal loss in the putamen. This treatment also resulted in a decrease in monomeric α-synuclein level in the striatum (Bassil et al., 2017). Currently, phase 2 of the trial is ongoing, which is assessing the efficacy of a weekly GLP-1 analog injection for 48 weeks in patients with early stage MSA (US National Library of Medicine, 2020b).

### Neuroprotection

#### Increasing Neurotrophic Factor Level

Decreased levels of neurotrophic factors such as glial derived neurotrophic factor (GDNF), brain derived neurotrophic factor (BDNF), and insulin-like growth factor 1 (IGF1) have been identified in human brains with multiple system atrophy and in mouse models (Ubhi et al., 2010). These are likely due to dysfunction of oligodendroglia cells and subsequent downregulation of neurotrophic factor production due to α-synuclein accumulation. Thus, the administration of these neurotrophic factors is a possible treatment strategy for neurodegenerative diseases (Allen et al., 2013). Studies on mice have shown that GDNF infusion alleviated the neuropathological process and improved MSA-like motor dysfunction (Ubhi et al., 2010). Also, fluoxetine administration, which elevated GDNF and BDNF levels in the transgenic mouse brain, improved motor skills (Ubhi et al., 2012). An ongoing phase 1 trial is currently investigating the clinical effects and safety of infusing the GDNF-gene directly into the putamen of patients with MSA (US National Library of Medicine, 2020c).

Studies on transgenic mice have recently shown that FTY720-Mitox, a derivative of the FTY72 drug approved for multiple sclerosis, increased oligodendroglia GDNF mRNA and protein. FTY720-Mitox acted by suppressing the downregulation of GDNF expression, which is seen in MSA brain. Simultaneously, FTY720-Mitox administration resulted in lowered α-synuclein aggregation in the spinal cord and reduced microglial activation in the cerebellum. These effects coincided with improved motor skills and sweat function, an indicator of dysautonomia. Furthermore, FTY720-Mitox also acted as mitochondria protector, which is extremely promising, taking into consideration the possible role of mitochondrial dysfunction in MSA pathogenesis (Vidal-Martinez et al., 2020).

#### Focus on Mitochondrial Dysfunction

Oxidative stress due to mitochondrial impairment plays a significant role in the pathogenesis of progressive supranuclear palsy. *In vitro* and *in vivo* studies showed that a high level of reactive oxygen species (ROS) and reactive nitrogen species (RNS) arise because of reduced cell antioxidative acting capacity, accelerated by α-synuclein aggregation (Scudamore and Ciossek, 2018). Uric acid (UA) has been shown to have strong antioxidative properties and reduce serum ROS/RNS levels. Patients with MSA have been observed to have a decreased UA serum level, which correlates with motor dysfunction (Sakuta et al., 2016). Recently, a phase 2 study with the UA prodrug inosine 5′-monophosphate has been completed. The study showed good tolerance and safety of this treatment. Inosine 5′-monophosphate administration resulted in a significant elevation of serum UA levels. In parallel, participants who received the treatment tended to perform better on cognitive function examinations, as shown by the mini-mental state examination (MMSE) and Montreal Cognitive Assessment (MoCA) scores when compared with the placebo group (Jung Lee et al., 2020). Nevertheless, further investigation is needed to assess the long-term efficacy of this treatment.

### Neuroinflammation

Myeloperoxidase is an enzyme that acts in neutrophiles, eosinophiles, and monocytes. It is involved in the production of reactive nitrogen and oxygen species and is a mediator of inflammatory processes in many diseases (Aratani, 2018). Studies on MSA human brains and MSA mouse models confirm a high myeloperoxidase (MPO) expression in brain areas of neurodegeneration. MPO inhibition reduces neuroinflammation, decreases activation of microglia in striatum, and improves motor skills in MSA animal models (Stefanova et al., 2012a). These promising discoveries have resulted in a growing attention on downregulating the MPO expression as a possible treatment target. Currently, an ongoing phase 3 placebo-controlled clinical trial is aiming to assess the efficacy of BHV-3241 (verdiperstat) administration in patients with MSA as measured by changes in the UMSARS score (US National Library of Medicine, 2019b). Potential novel therapeutic approaches for MSA are summarized in Table 2.

**Table 2:** Multiple system atrophy (MSA): Summary of emerging novel therapies.

**Type of action**	**Drug**	**Phase**	**Status**	**Outcome/end points**	**References**
Inhibition of a- synuclein aggregation	Anle 138b	Pre-Clin	Completed	PLP-hαSyn mice show motor improvement, preservation of dopaminergic neurons, decreased microglial activation	Houlden et al., 2001; Ahmed et al., 2019
	Anle 138b	I	Active		
	Sirolimus (oral)	II	Terminated	Difference in UMSARS score between placebo and treated group	Dickson, 1999
	EGCG	III	Completed	No improvement in the progression of the disease	Gilman et al., 2008
	Microtubule depolymerizing agent (Nocodazol)	Pre-Clin	Completed	Suppression of α-synuclein accumulation in mice models	Schapira, 2008
Immunization	PD01	Pre-Clin	Completed	Prevention of demyelination in neocortex and striatum, production of specific antibodies, motor improvement in transgenic mice	Surguchev and Surguchov, 2017
	PD01; PD03	I	Completed	Significant sustained antibody IgG response against PDO1 and PD03, therapy safe and well-tolerable	Watts et al., 2013; Coon and Singer, 2020
	anti-α-synuclein antibody	Pre-Clin	Completed	Suppression of α-synuclein intracellular accumulation in spinal cord and hippocampus, lowered GCIs concentration in oligodendroglia	Djelloul et al., 2015
Stem cell therapies	hMSC injection	Pre-Clin	Completed	Prevention of neuronal loss in striatum, motor improvement in hMSC treated mice	Burré, 2015; Brundin et al., 2017
	hMSC intraarterial/intravenous administration	II	Completed	Suppression of disease progression assessed by UMSARS score	Wagner et al., 2013
	autologous hMSC intravenous administration	I	Completed	Proved to be safe and well-tolerated	Heras-Garvin et al., 2019
	autologous hMSC intravenous administration	Phase I follow up	Active	Aims to conduct long term follow up of the patients who took part in phase I	US National Library of Medicine, 2020e
	intrathecal administration of hMSC	I/II	Completed	Proved to be safe and well-tolerated, suppression of disease progression assessed with UMSARS score	Kim and Guan, 2015
Addressing insulin resistance	Intranasal insulin	II	Completed	Improved cognitive and motor performance in treated patients	US National Library of Medicine, 2018
	Exendin-4 subcutanous administration	Pre-Clin	Completed	Lowered insulin resistance and decreased monomeric α-synuclein level in striatum	Lopez-Cuina et al., 2018
	GLP-1 analog	II	Active	Change in UMSARS score between placebo and treated group, assessment of safety and tolerability	Tanji et al., 2013
Increasing neurotrophic factor levels	GDNF infusion	Pre-Clin	Completed	Attenuation of motor deficits, alleviation of neuropathological process	Stefanova et al., 2012b
	Fluoxetine	Pre-Clin	Completed	Improvement of motor skills in transgenic mice and decreased neurodegeneration in neocortex and hippocampus	Mandler et al., 2015
	GDNF gene infusion into putamen	I	Active	Change in UMSARS score between placebo and treated group, incidence of treatment adverse effects and serious adverse effects, change in quality of life	Meissner et al., 2020
	FTY720-Mitoxy	Pre-Clin	Completed	Motor skills and sweat function improvement, lowered α-synuclein aggregation in spinal cord	US National Library of Medicine, 2014–2017b
Fighting oxidative stress (mitochondrial dysfunction)	inosine 5′-monophosphate	II	Completed	Improvement in cognitive function assessed with MoCA and MMSE in comparison with placebo group	Caplan and Dennis, 2006
Reducing neuroinflammation	MPO inhibitor	Pre-Clin		Improvement of motor skills in transgenic mice model concomitant with reduction of neuroinflammation and activation of microglia in striatum	Stemberger et al., 2011
	Verdiperstat	III		Change in UMSARS score between placebo and treated group, change in quality of life, assessment of safety and tolerability	Lee et al., 2012

## Discussion and Conclusions

Progressive supranuclear palsy, multiple system atrophy, and corticobasal degeneration, classified as atypical parkinsonian diseases, have only been treated symptomatically so far. Considering all of the recently published information about potential causes and clinical trials targeting candidates involved in disease development, one can hope that treatments addressing both disease symptoms and progression will lead to novel therapeutics and potential cures in the near future.

## Author Contributions

DP and WM: literature review and manuscript preparation. NM: study design, review of the manuscript, and final acceptance. All authors contributed to the article and approved the submitted version.

## Conflict of Interest

The authors declare that the research was conducted in the absence of any commercial or financial relationships that could be construed as a potential conflict of interest.

## Publisher's Note

All claims expressed in this article are solely those of the authors and do not necessarily represent those of their affiliated organizations, or those of the publisher, the editors and the reviewers. Any product that may be evaluated in this article, or claim that may be made by its manufacturer, is not guaranteed or endorsed by the publisher.

## Abbreviations

AD: Alzheimer's disease
ALCAR: acetyl-L-carnitine
AMPA: α-amino-3-hydroxy-5-methyl-4-isoxazolepropionic acid
AMPK: activated protein kinase
APS: atypical parkinsonian syndromes
ASOs: antisplicing oligonucleotides
ATF4: activating transcription factor 4
Aβ: amyloid β
BBB: blood brain barrier
BDNF: brain-derived neurotrophic factor
BFT: benfotiamine
BMSC: bone marrow stem cells
BtxA: botulinum toxin A
CBD: corticobasal degeneration
CBS: corticobasal syndrome
CDK5: cyclin-dependent kinase 5
CDK5: serine/threonine protein kinase 5
CML: chronic myeloid leukemia
CNS: central nervous system
COX-2: cyclooxygenase-2
CREB: cAMP response element binding
CSF: cerebrospinal fluid
CTE: chronic traumatic encephalopathy
CX3CR1: microglial specific fractalkine receptor
EGCG: epigallocatechin gallate
EIF2A: eukaryotic translation initiation factor
FBS: frontal behavioral-spatial syndrome
FTD: frontotemporal dementia
FTLD: frontotemporal lobar degeneration
GABA: γ-aminobutyric acid
GCI: glial cytoplasmic inclusions
GDNF: glial derived neurotrophic factor
GFAP: glial fibrillary acidic protein
GLP1: glucagon-like peptid 1 analog
GSK-3α: glycogen synthase kinase-3α
GSK-3α/3β: glycogen synthase kinase-3α and 3β
GSK-3β: glycogen synthase kinase-3β
hASCs: human adipose derived stem cells
Hsp70: heat shock protein 70
IGF1: insulin-like growth factor 1
IL-1R: interleukin-1 receptor
iPDEs: inhibitors of phosphodiesterases
5-LO: 5-lipooxygenase
LTs: leukotrienes
MAPT: microtubule-associated protein tau
MB: methylene blue
MMSE: Mini-Mental State Examination
MOBP –: myelin-associated oligodendrocyte basic protein
MPO: myeloperoxidase
MRI: magnetic resonance imaging
MSA-A: multiple system atrophy with dominant autonomic symptoms
MSA-C: multiple system atrophy with dominant cerebellar symptoms
MSA: multiple system atrophy
MSA-P: multiple system atrophy with dominant parkinsonian symptoms
MSC: mesenchymal stem cells
MTBD: microtubule-binding domain
NAD: nicotinamide adenine dinucleotide
NADP+: nicotinamide adenine dinucleotide phosphate
naPPA: nonfluent variant of primary progressive aphasia
NBMI: N,N'-bis (2-mercaptoethyl) isophthalamide
NfL: neurofilament light chain
NMDA: N-methyl-D-aspartic acid
NMSS: Non-Motor Symptoms Scale
NPC-: neural protein cells
Nrf2/ARE: NF-E2-related factor 2 (nuclear factor erythroid 2-related factor 2)/antioxidant responsive element
NSAIDs: nonsteroidal antiinflammatory drugs
O-GlcNAc: O-linked β-N-acetylglucosamine
O-GlcNAcase: OGA, β-N-acetylglucosaminidase
6-OHDA: 6-hydroxydopamine
p38-MAPK: p38-mitogen activated protein kinase
PD: Parkinson's disease
PERK: phosphorylated endoplasmic reticulum kinase
PET: positron emission tomography
PGRN: progranulin gene
PKA: cAMP-dependent protein kinase A
PP2A: protein phosphatase 2A
PSD-95: postsynaptic density protein-95
PSI: systemic proteasome suppression
PSP: progressive supranuclear palsy
PSPRS: Progressive Supranuclear Palsy Rating Scale
PSPS: progressive supranuclear palsy like syndrome
RA: retinoic acid
RNS: reactive nitrogen species
ROS: reactive oxygen species
4RT: primary four repeat tauopathies
siRNA: short-interfering RNA
sMAPT: symptomatic MAPT mutation carriers
TA: tolfenamic acid
TBI: traumatic brain injury
TES: traumatic encephalopathy syndrome
TLR-4: toll-like receptor 4
TNFα: tumor necrosis factor α
UA: uric acid
UMSARS: Unified MSA Rating Scale
UPR: unfolded protein response
UPS: ubiquitin-proteasome system
YKL-40: chitinase-3-like protein-1.
